# Gender Differences in Large-Scale and Small-Scale Spatial Ability: A Systematic Review Based on Behavioral and Neuroimaging Research

**DOI:** 10.3389/fnbeh.2019.00128

**Published:** 2019-06-18

**Authors:** Li Yuan, Feng Kong, Yangmei Luo, Siyao Zeng, Jijun Lan, Xuqun You

**Affiliations:** Shaanxi Provincial Key Laboratory of Behavior and Cognitive Neuroscience, School of Psychology, Shaanxi Normal University, Xi'an, China

**Keywords:** gender differences, large-scale spatial ability, small-scale spatial ability, activation likelihood estimation, meta-analysis

## Abstract

**Background:** As we human beings are living in a multidimensional space all the time. Therefore, spatial ability is vital for the survival and development of individuals. However, males and females show gender differences in this ability. So, are these gender differences influenced by the scale type of spatial ability? It's not well specified. Therefore, to tackle this issue, we conducted the current research from the behavioral and neural level.

**Methods:** Study 1 used the general meta-analysis method to explore whether individuals display the same gender differences in large- and small-scale spatial ability. Study 2 used the method of Activation Likelihood Estimation to identify the commonalities and distinctions of the brain activity between males and females on large- and small-scale spatial ability.

**Results:** Study 1 showed that in behavior performance, males outperformed females in both large-scale and small-scale spatial ability, but the effect size of the gender difference in large-scale spatial ability is significantly greater than that in small-scale spatial ability. In addition, Study 2 showed that in terms of neural activity, males and females exhibited both similarities and differences no matter in large-scale or small-scale spatial ability. Especially, the contrast analysis between females and males demonstrated a stronger activation in the brain regions of bilateral lentiform nucleus and bilateral parahippocampal gyrus in large-scale spatial ability, and correspondence in right sub-gyral, right precuneus, and left middle frontal gyrus in small-scale spatial ability.

**Conclusions:** The results indicated that the reason why females performed not so well in large-scale spatial ability was that they were more susceptible to emotions and their parahippocampal gyrus worked less efficiently than males; females performed not so well in small-scale spatial ability because they mostly adopted the egocentric strategy and their sub-gyral also worked less efficiently than males. The two different reasons have made for gender differences in favor of males in terms of spatial ability and such gender differences have different manifestations in large-scale and small-scale spatial ability. Possible implications of the results for understanding the issue of gender differences in spatial ability are discussed.

## Introduction

Gender differences have long been studied in a variety of fields like psychology and cognitive neuroscience. Gender differences in individual spatial ability have also been emphasized in the field of spatial ability. Our study aims to go deeper into gender differences in individual spatial ability through a meta-analysis based on behavioral performance and neural basis.

Spatial ability is one of the most core cognitive abilities of individuals. The tests of spatial ability also make up a significant part of intelligence testing. In general, spatial ability has been defined as the ability to understand the relationships among different positions in space or imagined movements of two- and three-dimensional objects (Clements, 1998; Wang et al., 2014). Specifically, space ability involves two major categories of large-scale spatial ability and small-scale spatial ability. Large-scale spatial ability refers to the ability of individuals to carry out cognitive processing of spatial information in the large-scale environment. And in this process, viewer's perspective changes with respect to the larger environment, but the spatial relationships among individual objects do not change (Hegarty and Waller, 2004; Wang et al., 2014). The most typical representatives of large-scale spatial ability are navigation ability and spatial orientation ability (Jansen, 2009; Tim and Höffler, 2010; Wang et al., 2014). The navigation ability refers to the ability to navigate in a large-scale environment where the spatial relationships among landmarks cannot be fully apprehended from a single vantage point (Wang and Carr, 2014). Spatial orientation is the ability to imagine objects from different perspectives (Yilmaz, 2009; Turgut, 2015). Small-scale spatial ability means the ability to mentally represent and transform two- and three-dimensional images, which can typically be apprehended from a single vantage point (Wang and Carr, 2014). Small-scale spatial ability mainly includes the capabilities of spatial visualization and spatial relations (Jansen, 2009; Tim and Höffler, 2010). Spatial visualization refers to the ability to manipulate complex spatial information involving configurations of shapes, such as image fold and movement, or changing their thinking of a two-dimensional object into three-dimensional one (Linn, 1985; Yang and Chen, 2010). Spatial relations ability means to recognize the relationships among visual components of an object (Bosnyák and Nagy-Kondor, 2008; Turgut, 2015).

It remains unclear in current studies whether individuals display similarities or same gender differences in large-scale and small-scale spatial ability. In fact, studies conflict with one another on gender differences in individual spatial ability; that is to say, there are three types of results whether studies concern large-scale or small-scale spatial ability: males show better spatial ability; females show better spatial ability; there is no gender difference in spatial ability (Rilea et al., 2004; Coluccia et al., 2007; Rilea, 2008; Gabriel et al., 2011; Hoffman et al., 2011; Burke et al., 2012). It is exactly because of these conflicting results that gender differences in spatial ability have long been insufficiently summarized or explained. One of the main purposes of our study is to make a meta-analysis of gender differences in individual spatial ability. On a behavioral level, this meta-analysis provides the following advantages: (1) Results can be generalized to a larger population, (2) The precision and accuracy of estimates can be improved as more data is used. This, in turn, may increase the statistical power to detect an effect. (3) Inconsistency of results across studies can be quantified and analyzed. Moderators can be included to explain variation between studies. The Activation Likelihood Estimation (ALE) method may focus on resolving the following problems in the current brain imaging research: firstly, the number of subjects in the single brain imaging study is generally less, and the results are not stable enough; secondly, the result of a single neuroimaging are probably affected by certain experimental operations (e.g., scan parameters); thirdly, the interpretation of the function of a certain brain region by a single brain imaging study is often limited to a single or a few tasks used.

It is noteworthy that there are already other meta-analyses of gender differences in individual spatial ability. For instance, Linn (1985) used the method of meta-analysis to investigate the questions on the sex differences in spatial ability. Their results suggested that firstly, sex differences arise on mental rotation and spatial perception, but not spatial visualization; secondly, large sex differences are found only on measures of mental rotation; thirdly, smaller sex differences are found on measures of spatial perception; finally, when sex differences are found, they can be detected across the life span. Voyer et al. (1995)'s meta-analysis found that sex differences, favoring males, are clearly significant and homogeneous on the Cards Rotation Test, the generic mental rotation task, the Spatial Relations task, and the Paper Form Board task. Sex differences on the Spatial Relation task and Paper Folding are homogeneous but not significant. The rod and frame test and the Block Design subtest of the various Wechsler intelligence scales show sex differences in some age groups but not others. Finally, scoring and testing procedures proved to have an important influence on the magnitude of sex differences on the Mental Rotations Test, the Water Level Test, the Identical Blocks Test, and the Embedded Figures Test. The size of the failsafe numbers associated with the different analyses suggests that the file drawer phenomenon is not sufficient to account for the prevalence of significant sex differences. Maeda and Yoon (2013) conducted a meta-analysis to estimate the magnitude of gender difference in mental rotation ability and to investigate how factors related to test administration conditions play a role in varying gender difference effect sizes and threatening validity. The results indicated that male participants outperformed females on the test. And the moderator analysis indicated that male superiority on spatial ability tasks is related to the implementation of time limits. The gender difference became larger when stringent time limits were implemented. Reilly and Neumann (2013)'s results of meta-analysis showed that measuring tools can influence gender differences in the studies of individual mental rotation ability. Specifically, Vandenberg instrument produced the highest gender-role effect size of any mental rotation task.

Overall, these meta-analyses show that men are significantly better than women in spatial ability and that such gender difference is subject to measuring tool, task type, experimental time limit, and other factors. It is a pity that they are mostly focused on gender differences in small-scale spatial ability (particularly mental rotation ability), with little reference to gender differences in spatial ability at different scales and the neural basis of such gender differences that are crucial to a comprehensive grasp of gender differences in individual spatial ability. Our meta-analysis attempts to explore whether individuals display the same gender differences in large-scale and small-scale spatial ability and answer “What are the manifestations of such gender differences?,” “What are the contributing factors?,” and “What is the neural basis?”

## Study 1

Study 1 discussed gender differences in individual spatial ability on a behavioral level. A moderation analysis was performed of the region, age and education level of the subjects and the time of research publication (all of which are claimed to be big individual spatial ability-influencing factors by existing studies; Silverman et al., 2007; Hoffman et al., 2011; Techentin et al., 2014; Pietschnig and Gittler, 2015) as well as the type of spatial ability.

## Methods

### Literature Search

We searched the studies on the subject of “spatial ability” published in the past 20 years (1988.01–2018.06) on the database retrieval platform (Web of Science, PubMed, PsycINFO, Google Scholar, and CNKI). And, in order to collect the target documents to the maximum extent, we classified the search keywords into the following four series according to the concept and structure of spatial ability, a total of 38 groups:

1. Spatial ability; Spatial cognition; Spatial perception; Spatial information processing.
2. Large-scale Spatial ability; Small-scale Spatial ability.
3. Navigation; Spatial orientation; Spatial visualization; Spatial relations.
4. Navigation task/ test; Draw maps task/ test; Way-finding task/ test; Map learning task/ test; Spatial orientation task/ test; Perspective taking task/ test; Spatial visualization task/ test; Mental rotations task/ test; Paper folding task/ test; Spatial relations task/ test; Water level task/ test; Card rotation task/ test; Figures task/ test; Differential aptitude task/ test.

### Inclusion and Exclusion Criteria

After four rounds of the above-mentioned search, a total of 1,714 documents were obtained. Then we examined each of the documents in full and incorporated the documents with the following characteristics into the meta-analysis of this study:

1. All of the subjects are sample groups of healthy people.
2. The specific experimental method in the study must be behavioral. And the articles must include reports of the males' and females' performance (Means ± Standard deviation, M ± SD, etc.), or statistical test results (r, t, or effect size, etc.) which related to the gender difference, when they finish their independent experimental task respectively.
3. If an experimental result is reported many times in multiple papers, but it has been recorded only once in the meta-analysis, the research data cannot be used again.

**Figure 1:**
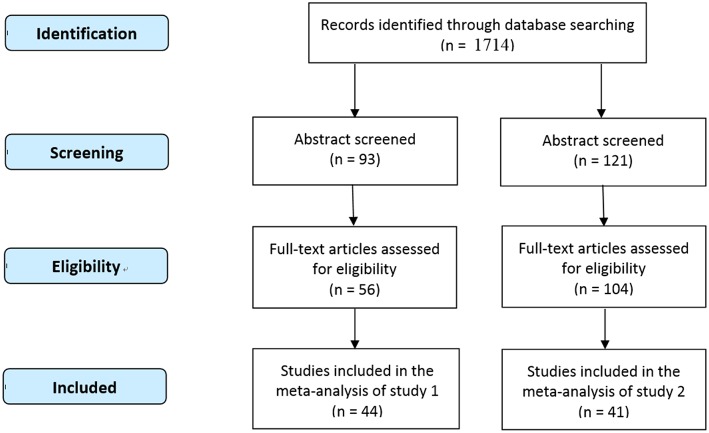
Procedure of data selection [PRISMA 2009 Flow Diagram; Moher et al. (2009); http://www.prisma-statement.org].

After the above screening process, there were totally 44 studies (see Table 1;, Figure 1), involving 18,522 participants (male = 8,424), that met the standards, and a total of 98 effect size were incorporated in the meta-analysis of this research. Among them, there were 14 effect size associated with large-scale spatial ability and 84 effect size associated with small-scale spatial ability.

**Table 1:** Summary of studies included in the present meta-analyses of study 1.

**Study**	***N* (male)**	**Age**	**Region**	**Identity**	**Year**	**Indicator**	**Spatial ability type**	**g**
Alexander, 2006^u1^	64 (29)	20	USA	Undergraduate	2006	Score	Small-scale	1.19
Alexander, 2006^u2^	64 (29)	20	USA	Undergraduate	2006	Score	Small-scale	−0.98
Allison et al., 2017	72 (35)	21.62	UK	Undergraduate	2017	Time	Large-scale	4.9
Battista, 1990	126 (53)	Non	USA	Middle school student	1990	Score	Small-scale	0.94
Brownlow et al., 2003	48 (32)	Non	USA	Undergraduate	2003	Score	Small-scale	−0.08
Black, 2005^u1^	97 (64)	24.42	USA	Non	2005	Score	Small-scale	0.38
Black, 2005^u2^	97 (64)	24.42	USA	Non	2005	Score	Small-scale	0.4
Black, 2005^u3^	97 (64)	24.42	USA	Non	2005	Score	Small-scale	0.04
Burke et al., 2012	185 (80)	17	UK	Non	2012	Time	Large-scale	2.58
Cai and Chen, 2000^a^	39 (25)	Non	China	Pupil	2000	Score	Small-scale	0.29
Cai and Chen, 2000^b^	35 (15)	Non	China	Pupil	2000	Score	Small-scale	0.57
Cai and Chen, 2000^c^	46 (31)	Non	China	Middle school student	2000	Score	Small-scale	0.94
Cai and Chen, 2000^d^	55 (26)	Non	China	Middle school student	2000	Score	Small-scale	0.64
Cai and Chen, 2000^e^	37 (19)	Non	China	Middle school student	2000	Score	Small-scale	0.29
Cai and Chen, 2000^f^	48 (26)	Non	China	Middle school student	2000	Score	Small-scale	1.47
Cai and Chen, 2000^g^	38 (5)	Non	China	Middle school student	2000	Score	Small-scale	−0.29
Cai and Chen, 2000^h^	45 (15)	Non	China	Middle school student	2000	Score	Small-scale	0.38
Cai and Chen, 2000^i^	42 (16)	Non	China	Middle school student	2000	Score	Small-scale	0.08
Cai and Chen, 2000^j^	34 (8)	Non	China	Middle school student	2000	Score	Small-scale	0.99
Cai and Chen, 2000^k^	63 (34)	Non	China	Undergraduate	2000	Score	Small-scale	0.77
Colom et al., 2004	1593 (794)	Non	Spain	Graduate	2004	Time	Large-scale	1.02
Contreras et al., 2007	2624 (1291)	28	Spain	Graduate	2007	Time	Large-scale	−0.59
Coluccia et al., 2007	96 (48)	22.31	Italy	Undergraduate	2007	Score	Large-scale	0.35
Evardone and Alexander, 2009	105 (50)	20.2	USA	Non	2009	Score	Small-scale	1.05
Fields and Shelton, 2006^u1^	40 (20)	Non	USA	Undergraduate	2006	Score	Large-scale	0.52
Fields and Shelton, 2006^u2^	40 (20)	Non	USA	Undergraduate	2006	Score	Small-scale	1.23
Fields and Shelton, 2006^u3^	40 (20)	Non	USA	Undergraduate	2006	Score	Large-scale	1.07
Fields and Shelton, 2006^u4^	40 (20)	Non	USA	Undergraduate	2006	Score	Large-scale	0.03
Feng and Tian, 2009	81 (20)	Non	China	Undergraduate	2009	Score	Small-scale	0.26
Gabriel et al., 2011^u1^	74 (40)	20.5	Canada	Undergraduate	2011	Accuracy	Small-scale	0.48
Gabriel et al., 2011^u2^	74 (40)	20.5	Canada	Undergraduate	2011	Time	Small-scale	0.79
Ganley and Vasilyeva, 2011	114 (67)	14	USA	Middle school student	2011	Score	Small-scale	0.62
Ganley et al., 2014	113 (66)	14	USA	Middle school student	2014	Score	Small-scale	0.6
Hou et al., 1998^a^	30 (15)	3.75	China	Kindergarten	1998	Score	Small-scale	−0.09
Hou et al., 1998^b^	24 (12)	4.25	China	Kindergarten	1998	Score	Small-scale	−0.47
Hou et al., 1998^c^	19 (11)	4.75	China	Kindergarten	1998	Score	Small-scale	−0.19
Hou et al., 1998^d^	22 (10)	5.25	China	Kindergarten	1998	Score	Small-scale	0.11
Hou et al., 1998^e^	21 (10)	5.75	China	Kindergarten	1998	Score	Small-scale	0.24
Hou et al., 1998^f^	27 (13)	6.25	China	Kindergarten	1998	Score	Small-scale	0.1
Hegarty et al., 2006^u1^	218 (135)	22	USA	Mixed	2006	Score	Small-scale	0.21
Hegarty et al., 2006^u2^	218 (135)	22	USA	Mixed	2006	Score	Small-scale	0.74
Hegarty et al., 2006^u3^	218 (135)	22	USA	Mixed	2006	Score	Large-scale	0.2
Heyden et al., 2016^au1^	210 (76)	9.92	Netherlands	Pupil	2016	Score	Small-scale	0.31
Heyden et al., 2016^au2^	210 (76)	9.92	Netherlands	Pupil	2016	Score	Small-scale	0.41
Heyden et al., 2016^bu1^	152 (49)	12	Netherlands	Pupil	2016	Score	Small-scale	−0.08
Heyden et al., 2016^bu2^	152 (49)	12	Netherlands	Pupil	2016	Score	Small-scale	−0.03
Hromatko and Butkovic, 2009^u1^	201 (51)	20.81	Vienna	Undergraduate	2009	Score	Small-scale	0.74
Hromatko and Butkovic, 2009^u2^	201 (51)	20.81	Vienna	Undergraduate	2009	Score	Small-scale	0.64
Hoffman et al., 2011	1,279 (661)	18	India	Non	2011	Time	Small-scale	0.19
Jansen and Heil, 2009	50 (25)	22.8	Germany	Non	2009	Score	Small-scale	3.79
Kaufman, 2007^u1^	100 (50)	17	UK	Graduate	2007	Score	Small-scale	1.16
Kaufman, 2007^u2^	100 (50)	17	UK	Graduate	2007	Score	Small-scale	0.42
Lj and Borst, 2011^u1^	75 (37)	23	USA	Graduate	2011	Score	Small-scale	0.43
Lj and Borst, 2011^u2^	75 (37)	23	USA	Graduate	2011	Score	Small-scale	0.12
Langlois et al., 2013^a^	59 (42)	Non	Canada	Graduate	2005	Score	Small-scale	0.86
Langlois et al., 2013^b^	54 (43)	Non	Canada	Graduate	2006	Score	Small-scale	1.07
Langlois et al., 2013^c^	18 (9)	Non	Canada	Graduate	2007	Score	Small-scale	0.36
Langlois et al., 2013^d^	34 (12)	Non	Canada	Graduate	2008	Score	Small-scale	0.28
Langlois et al., 2013^e^	49 (25)	Non	Canada	Graduate	2010	Score	Small-scale	0.78
Langlois et al., 2013^f^	59 (42)	Non	Canada	Graduate	2005	Score	Small-scale	0.68
Langlois et al., 2013^g^	54 (43)	Non	Canada	Graduate	2006	Score	Small-scale	1.45
Langlois et al., 2013^h^	18 (9)	Non	Canada	Graduate	2007	Score	Small-scale	0.22
Langlois et al., 2013^i^	34 (12)	Non	Canada	Graduate	2008	Score	Small-scale	0.16
Langlois et al., 2013^j^	49 (25)	Non	Canada	Graduate	2010	Score	Small-scale	0.91
Liu, 2016	80 (35)	5.5	China	Kindergarten	2016	Score	Small-scale	0.21
Liao and Dong, 2017	20 (10)	23	China	Graduate	2017	Time	Large-scale	0.69
Maeda and Yoon, 2015	2468 (580)	Non	USA	Undergraduate	2015	Score	Small-scale	0.6
Merrill et al., 2016^u1^	153 (73)	17.53	USA	Middle school student	2016	Time	Large-scale	2.9
Merrill et al., 2016^u2^	153 (73)	17.53	USA	Middle school student	2016	Time	Small-scale	0.14
Merrill et al., 2016^u3^	153 (73)	17.53	USA	Middle school student	2016	Time	Small-scale	0.37
Merrill et al., 2016^u4^	153 (73)	17.53	USA	Middle school student	2016	Time	Small-scale	0.07
Poulin et al., 2004	218 (132)	Non	USA	Undergraduate	2004	Score	Small-scale	7.71
Ritter, 2004	79 (37)	21	UK	Non	2004	Score	Small-scale	0.73
Rilea et al., 2004^u1^	105 (53)	19.55	USA	Undergraduate	2004	Time	Small-scale	1.12
Rilea et al., 2004^u2^	105 (53)	19.55	USA	Undergraduate	2004	Time	Small-scale	4.58
Rilea et al., 2004^u3^	105 (53)	19.55	USA	Undergraduate	2004	Time	Small-scale	0.77
Ruggiero et al., 2008	60 (30)	23.5	Italy	Undergraduate	2008	Score	Small-scale	0.56
Signorella et al., 1989^a^	344 (146)	Non	USA	Non	1989	Score	Small-scale	0.53
Signorella et al., 1989^b^	288 (132)	Non	USA	Non	1989	Score	Small-scale	0.59
Samsudin, 2008	33 (13)	15.5	Malaysia	Middle school student	2008	Score	Small-scale	0.33
Tuttle and Pillard, 1991	150 (49)	30.8	USA	Non	1991	Score	Small-scale	0.61
Tao et al., 2008	260 (125)	14.66	China	Middle school student	2008	Score	Small-scale	0.58
Tzuriel and Egozi, 2010^u1^	116 (58)	6.5	Israeli	Kindergarten	2010	Score	Small-scale	0.27
Tzuriel and Egozi, 2010^u2^	116 (58)	6.5	Israeli	Kindergarten	2010	Score	Small-scale	0.83
Wu and Yang, 2014^u1^	588 (290)	16.92	China	Middle school student	2014	Score	Large-scale	0.22
Wu and Yang, 2014^u2^	588 (290)	16.92	China	Middle school student	2014	Score	Small-scale	0.43
Wu and Yang, 2014^u3^	588 (290)	16.92	China	Middle school student	2014	Score	Small-scale	0.13
Xu et al., 2016^a^	1173 (570)	Non	USA	Undergraduate	2016	Score	Small-scale	0.8
Xu et al., 2016^b^	1110 (537)	Non	USA	Undergraduate	2016	Score	Small-scale	0.61
Xu et al., 2016^c^	1175 (568)	Non	USA	Undergraduate	2016	Score	Small-scale	0.58
Xu et al., 2016^d^	1089 (528)	Non	USA	Undergraduate	2016	Score	Small-scale	0.74
Yonker et al., 2008	128 (78)	69.4	Sweden	Mixed	2008	Score	Small-scale	4.05
Yu et al., 2008^u1^	44 (22)	22.6	China	Undergraduate	2008	Score	Small-scale	−1.08
Yu et al., 2008^u2^	44 (22)	22.6	China	Undergraduate	2008	Score	Small-scale	−0.63
Zacks et al., 2001^u1^	48 (22)	Non	USA	Undergraduate	2001	Score	Small-scale	4.09
Zacks et al., 2001^u2^	48 (22)	Non	USA	Undergraduate	2001	Score	Large-scale	3.37
Zacks et al., 2001^u3^	48 (22)	Non	USA	Undergraduate	2001	Score	Large-scale	1.91
Zhou and Yun, 2011	20 (10)	Non	China	Undergraduate	2011	Score	Small-scale	−0.27

u1–u4 *Studies report more than one result for each independent sample*.

a–k *Studies include more than one independent sample*.

### Literature Coding

Literature coding is carried out by two researchers separately. The coding results will be compared and, if there is any inconsistency, will be decided by all researchers through discussion. In our study, two researchers produced highly (96%) consistent coding results. The coding content is as follows: (1) authors of literature; (2) numbers of male and female subjects in the experiment; (3) behavioral experimental scores of males and females; (4) type of indicators and direction of gender advantage; (5) type of spatial ability tested in the experiment; (6) region of the subjects; (7) age of the subjects; (8) education level of the subjects; (9) year of publication. (5)–(9) are the moderator variables to be tested in our study.

### Effect Size Computation

Our study adopted CMA2.0 for meta-analysis. For continuous data with different units, Cohen's d is often used as a measure of effect in meta-analysis (Cohen, 1988). In studies with varying sample sizes, Cohen's d may produce biased effect estimates. In a study with a small sample size, for example, the effect estimate may be biased to higher values. To correct this, Hedges proposed Hedges' g for an unbiased estimate of effect size (Borenstein et al., 2009; Card, 2012). The corrected, standardized mean difference Hedges' g was used in our study as an unbiased effect size.

### Heterogeneity Test

A heterogeneity test must be conducted before measuring the total effect and the moderator effect to see if there is a statistical difference between studies. Cochran's *Q* test is often used to identify heterogeneity. If the test yields a *p* value ≤ to 0.01, it can be taken as evidence of heterogeneity; otherwise, it means homogeneity. In addition, *I*^2^ provides a measure of the degree of heterogeneity. A higher *I*^2^ value suggests a higher degree of heterogeneity. For example, 25, 50, and 75% mean low, moderate and high heterogeneity, respectively.

### Model Selection

There are mainly two models for meta-analysis: fixed effects and random effects models; their biggest difference lies in weight. The fixed effects model assumes that there is only one true effect size behind all studies and that the effect size difference between studies is caused by the sampling error. Its meta-analytical results don't apply to other non-meta-analytical studies. The random effects model holds that the true effect size varies between studies and that the effect size difference between studies is due to the true effect size difference as well as the sampling error. With the sample difference taken into consideration, the random effects model's results apply to a broader scope of studies. A heterogeneity test can be used to help model selection. A random effects model will be more suitable in the case of heterogeneity in total effect sizes (Borenstein et al., 2009).

### Evaluation of Publication Bias

Publication bias occurs when statistically significant studies are more likely to be published than statistically insignificant studies, adding to the difficulty of collecting statistically insignificant literature during meta-analysis, leading to a systematic error between the included studies and the actual studies, and finally affecting the results of meta-analysis. A host of methods are available for publication bias detection. In our study, a funnel plot, Rosenthal's *Failsafe N* and Egger's Regression Intercept were employed to detect publication bias.

## Results

### Heterogeneity Results

Our study first made a heterogeneity test of total effect sizes to decide whether to use a fixed effects model or a random effects model for data analysis. The results are shown in Table 2. The *Q-*value was significant (*p* < 0.001), indicating that the effect sizes were heterogeneous. The *I*^2^ value was 95.67, indicating that 95.67% of total variation came from the true difference between effect sizes in the model while only 4.33% came from the random error. As 25, 50, and 75% stand for low, moderate, and high heterogeneity, the *I*^2^ value of 95.67 indicates high heterogeneity among effect sizes in our study. In view of this, a random effects model was adopted in the analysis of the total effect and the moderator effect. Meanwhile, the *Tau*^2^ value was 0.47, indicating that 47% of total variation across studies can be used for calculating weight when putting weight on various studies in the random effects model.

**Table 2:** The results of total effect and heterogeneity test.

**k**	**g**	**95% CI**	**Q**	**df**	***I*^2^**	**Tau^2^**
101	0.72^***^	[0.58, 0.86]	2309.14^***^	100	95.67	0.47

k is the number of independent effect size; n is the sample size; g is the Hedges's g; CI, confidence interval;

*** *p < 0.001*.

### Publication Bias Results

**Figure 2:**
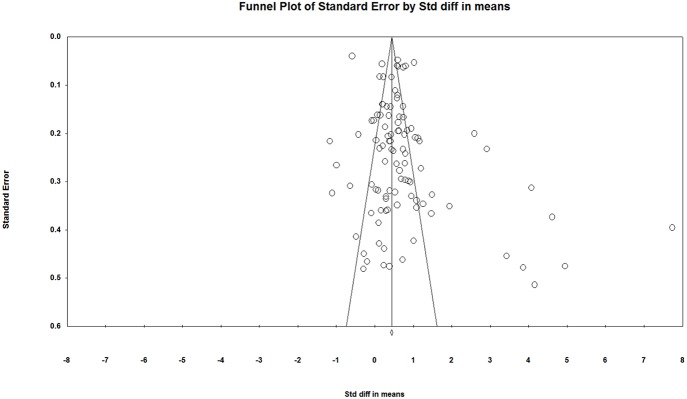
Funnel plot of standard error by standard difference in means.

A funnel plot, Rosenthal's *Failsafe N* and Egger's Regression Intercept were used to detect publication bias in our study. According to the funnel plot (Figure 2), the studies were not evenly distributed on both sides of the total effect size with more on the right side, suggesting a likely presence of publication bias in current studies on individual spatial ability. The funnel plot was only a preliminary look at publication bias from a subjective perspective. In order to take a comprehensive look, we performed the tests of Rosenthal's *Failsafe N* and Egger's Regression Intercept. In terms of Rosenthal's *Failsafe N*, if the N*fs* value is <5*k*+10, it is a reminder of the potential impacts of publication bias on current studies^24^. In our study, the N*fs* value was 7,057, larger than the critical value of 5*k*+10 (515), suggesting an unlikely presence of publication bias in current studies. As for Egger's Regression Intercept, if the regression intercept is close to 0, it is less likely that there is a publication bias. In our study, the intercept value was 2.06, *p* < 0.05, 95% confidence interval (CI) is [0.61, 3.52], suggesting that there may still be a slight publication bias in current studies. Two of the three publication bias tests showed evidence of a possible publication bias, and therefore we concluded that there was a slight publication bias in current studies. According to Borenstein et al. (2009), however, the purpose of a publication bias test is not to find if there is a publication bias in a meta-analysis but to assess if the publication bias impacts the reliability of the meta-analysis. To this end, Borenstein et al. (2009) provided three results of publication bias assessment: first, the impact of bias is negligible; second, the impact of bias is non-negligible but the results are still valid; third, the results may be wrong. Considering that publication bias can't be completely avoided in psychology research since studies with negative results are less likely to be published, and given that the publication bias in our study isn't serious, we believe our meta-analytical conclusions still provide a reference point.

### Total Effect

**Figure 3:**
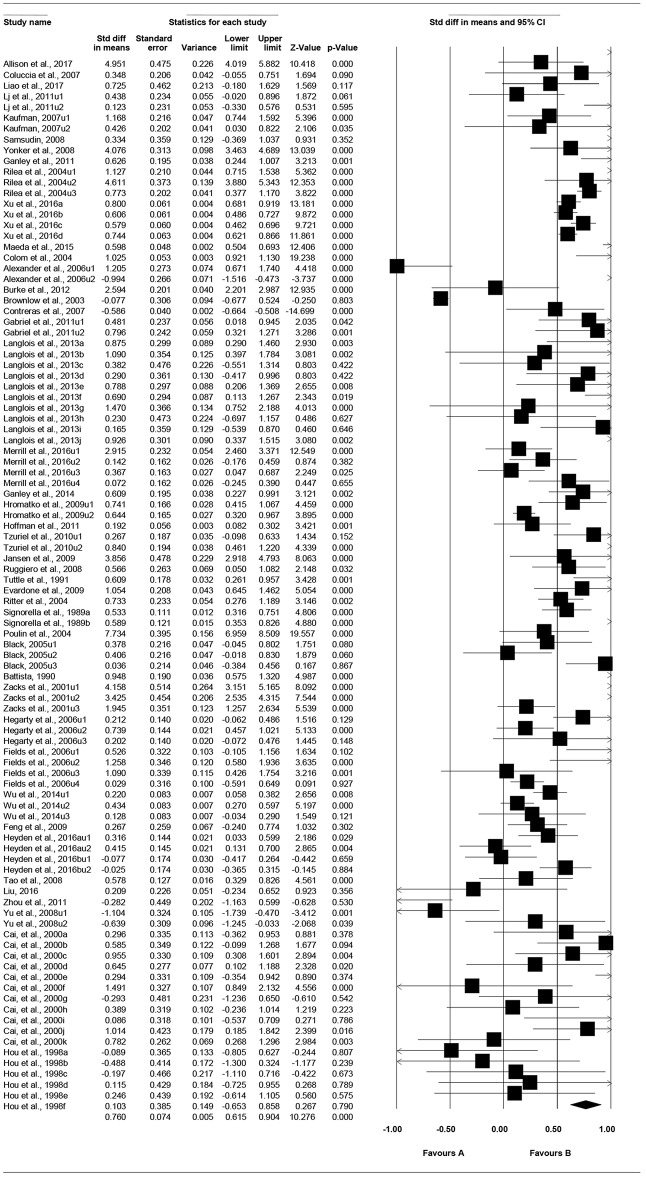
Forest plot under random-effects model.

As shown in Table 2, the total effect size *g* is 0.72, *p* < 0.001, 95% CI is [0.58, 0.86]. Figure 3 presents the “forest plot,” a graphic description of the results based on the random-effects modeling analysis of the all effect sizes. In this forest plot, each effect size (square dot) and its estimated confidence interval (horizontal lines extending from both sides of the squared dot) was graphically shown (Fan et al., 2017).

### Moderator Analyses Results

The results are shown in Table 3. The large-scale spatial ability group and the small-scale spatial ability group had a significant difference in effect size (Q_*between*_ = 5.25, *p* < 0.05), indicating that the type of individual spatial ability has a significant moderator effect, or that gender differences in spatial ability are subject to the type of spatial ability. To be specific, the effect size of gender differences in the large-scale spatial ability group reached a high level (*g* = 1.34, *p* < 0.001); the small-scale spatial ability group only reached a medium level (*g* = 0.62, *p* < 0.001). The heterogeneity in both groups also reached a high level (*I*^2^_*large*−*scale*_ _*spatial*_ _*ability*_ = 98.81%, *I*^2^_*small*−*scale*_ _*spatial*_ _*ability*_ = 92.19%).

**Table 3:** Moderator analyses.

**Moderator**	**k**	**g**	**95% CI**	**I^2^**	**Q_**between**_**
**Type of spatial ability**					5.25^*^
Large-scale spatial ability	14	1.34^***^	[0.74, 1.94]	98.81	
Small-scale spatial ability	87	0.62^***^	[0.49, 0.75]	92.19	
**Region**					35.77^***^
Europe	17	1.18^***^	[0.65, 1.70]	98.59	
America	50	0.90^***^	[0.73, 1.08]	93.74	
Asia	31	0.29^***^	[0.16, 0.43]	65.96	
**Age**					36.32^***^
0–5	6	−0.05	[−0.38, 0.27]	0	
6–10	5	0.41^***^	[0.21, 0.60]	40.65	
11–15	5	0.34^*^	[0.03, 0.65]	77.40	
16–20	18	0.89^***^	[0.55, 1.23]	96.41	
21–25	20	0.64^***^	[0.33, 0.95]	90.52	
Above 26	3	1.34	[−0.69, 3.38]	99.22	
**Education level**					31.31^***^
Undergraduate	34	0.96^***^	[0.7, 1.21]	96.11	
Graduate	17	0.6^*^	[0.11, 1.08]	97.63	
Middle school student	20	0.59^***^	[0.36, 0.83]	88.97	
Pupils	6	0.21^*^	[0.02, 0.41]	39.88	
Kindergarten	9	0.21	[−0.06, 0.48]	44.15	
**Year (Undergraduate)**					18.39^**^
1989–1995	None				
1996–2000	4	2.54^**^	[1.04, 4.03]	93.85	
2001–2005	5	2.82^*^	[0.54, 5.09]	98.87	
2006–2010	13	0.31	[−0.07, 0.68]	86.36	
2011–2015	4	0.56^***^	[0.32, 0.79]	36.54	
2016–2019	5	1.01^***^	[0.69, 1.32]	95.54	
**Year (Graduate)**					19.80^***^
1989–1995	None				
1996–2000	None				
2001–2005	3	1.01^***^	[0.91, 1.11]	0	
2006–2010	11	0.57^*^	[−0.01, 1.15]	94.47	
2011–2015	2	0.28	[−0.04, 0.60]	0	
2016–2019	1	0.73	[−0.18, 1.63]	0	
**Year (Middle school student)**					9.15^*^
1989–1995	1	0.95^***^	[0.58, 1.32]	0	
1996–2000	8	0.59^**^	[0.23, 0.96]	58.57	
2001–2005	None				
2006–2010	2	0.55^***^	[0.32, 0.79]	0	
2011–2015	5	0.35^***^	[0.18, 0.53]	69.59	
2016–2019	4	0.86	[−0.23, 1.95]	97.49	

When a g has asterisks, it means a significant sex difference; when a Q has asterisks, it means a significant moderating effect;

* p <0.05,

** p <0.01,

*** *p <0.001*.

In addition, different regional groups had a significant difference in effect size (*Q*_*between*_ = 35.77, *p* < 0.001), and so did different age groups (*Q*_*between*_ = 36.32, *p* < 0.001), different educational groups (*Q*_*between*_ = 31.31, *p* < 0.001), and different research time groups (*Q*_*between*_ _(*undergraduate*)_ = 18.39, *p* < 0.01; *Q*_*between*_ _(*graduate*)_ = 19.80, *p* < 0.001; *Q*_*between*_ _(*middle*_ _*school*_ _*student*)_ = 9.15, *p* < 0.05). Arguably, these four factors have a significant moderator effect; that is to say, gender differences in individual spatial ability are subject to the region, age and education of the subjects as well as the time of research. As for other groups, the effect size *g* and heterogeneity *I*^2^ are shown in Table 3. It is important to note here that all studies in the database were included in the data analysis when the current study examined the moderator effects of regional factor, age factor, and educational factor. However, when we tested the moderator effect of research time factor, we conducted independent data analysis for the database of undergraduates, graduates, and middle school students. The reason we do this is because current research wants to control as much as possible the interference of age and educational factors on this test process. The above-mentioned process of comparing the spatial ability differences of the tested participants with similar ages and education levels in different time studies is more effective in explaining this problem.

### Correlation Analysis Results

Besides, the current research further explores whether the above factors are more closely related to the gender differences in individual spatial ability based on the above findings. Among them, the type of spatial ability and regional factor are categorical variables, age factor, educational factor, and research time factor are grade variables. Therefore, we only analyzed the correlation between the last three factors and the gender differences in individual spatial ability. The results showed that there were significant positive correlations between age and gender differences in spatial ability (*r*_*age*_ = 0.519, *p* < 0.05) and education level and gender differences in spatial ability (*r*_*education*_ _*level*_ = 0.949, *p* < 0.05). There was no significant correlation between research time and gender differences in spatial ability.

## Discussion 1

Our study found that males significantly outperformed females in spatial ability overall, which is consistent with the findings of previous meta-analyses. In spite of some conflicting conclusions on gender differences in individual spatial ability, males are obviously at an advantage over females in spatial ability from a macro perspective, and this gender advantage remains obvious and stable, at least from the perspective of meta-analysis. The gender advantage, however, isn't obvious and stable enough to be free from any moderator effects. Our study has provided relevant evidence.

In particular, our study has discussed for the first time the type of spatial ability as a moderator between gender differences in individual spatial ability. Indeed, males outperformed females in both large-scale and small-scale Spatial Ability, but the gender differences were much different—the gender difference in large-scale spatial ability is significantly greater than that in small-scale spatial ability with the former at a high level and the latter at a medium level. Such findings provide a broader insight into gender differences in individual spatial ability.

Our study also found that males and females in different regions performed differently in spatial ability. The results of this study and the findings of Silverman et al. (2007)'s study have shown consistency to some extent. Silverman collected data on spatial ability test of nearly 250,000 participants in 40 countries through a network survey conducted by the BBC. The data show that individuals in different countries show significant gender differences in spatial ability. However, this gender difference did not show significant correlation with the Gross National Income (GNI) and Socioeconomic status (SES) indices. In fact, the biggest difference between the research included in the current research database and the Silverman et al. (2007)'s study is that Silverman adopts a non-laboratory design, while other studies adopt a laboratory research design. Therefore, from a more rigorous level, the effect sizes of these two types of experimental designs are not comparable. However, what is certain is that both the current study and Silverman et al. (2007)'s study indicated a regional difference of gender differences in individual spatial ability. Moreover, the current study also found that the gender differences in individual spatial ability in Europe and the Americas are larger, while in Asia, this gender difference is smaller. This is a very interesting discovery, but the existing data does not support a deeper discussion of the reasons for this phenomenon. Of course, we also believe that that the causes of this regional difference can be multifaceted, interactional, complicated, unstable, and remain to be examined in more studies.

In addition, age was found to be one of the biggest contributing factors to gender differences in individual spatial ability, which is also a good supplement to Techentin et al. (2014)'s meta-analytical findings. Their meta-analysis revealed a large age-related decrease in spatial performance on psychometric tests. Specifically, older adults performed worse on psychometric tests than younger adults. On this basis, we performed a correlation analysis of gender differences and age and found that gender differences in individual spatial ability increased as individuals grew older. Due to the complex interaction of other factors like individual differences, growth environment and experimental conditions, this finding may not apply to all meta-analyses, but the high correlation index represents and explains the development and growth trend in connection to gender differences in individual spatial ability.

Our study also verified Hoffman et al. (2011)'s findings about education as a contributing factor to gender differences in individual spatial ability. Our study found that different educational groups had significant gender differences in individual spatial ability. To be specific, gender differences in individual spatial ability increase with the education level from kindergarten to primary school, middle school, college, and postgraduate education. It is interesting that education seems to have widened the gender gap in individual spatial ability instead of making it up. How and why that happens may be complicated and at least unexplainable in our study since age was included as a factor into our individual education level-related analysis.

Our study finally examined the moderator effect of research time on gender differences in individual spatial ability though no connection was found between them. Some studies claim that spatial ability, a part of individual intelligence, is also subject to the Flynn effect. The Flynn effect is the substantial and long-sustained increase in both fluid and crystallized intelligence test scores measured in many parts of the world over the Twentieth century. And, the test score increases have been continuous and approximately linear from the earliest years of testing to the present (Pietschnig and Gittler, 2015). However, after comparing Pietschnig and Gittler (2015)'s findings with ours, we decided the Flynn effect was confined to the repeated test of individual spatial ability and that gender differences in individual spatial ability wouldn't change regularly with research time.

## Study 2

Study 1 summed up the different manifestations of individual gender differences in large-scale and small-scale Spatial Ability on the behavioral level. Is there a neural basis of such different manifestations? This is exactly the question explored in Study 2 where the ALE approach was used to discuss gender differences in large-scale and small-scale Spatial Ability on the level of neural basis.

In fact, there have been many studies on the causes of gender differences in individual spatial ability. For example, Lawton (1994), Malinowski and Gillespie (2001), Lawton and Kallai (2002), and Gabriel et al. (2011)'s studies all point out that the main reason for the gender difference in individual spatial ability is that females are more prone to spatial anxiety when performing spatial cognitive tasks. In addition, after a systematic review of all previous studies on gender differences in spatial abilities, Wang and Carr (2014) also proposed that the main reason for gender differences in spatial ability was that females and males used different cognitive strategies in spatial cognitive tasks. From the perspective of behavioral level, whether spatial anxiety or cognitive strategy, they have become the most commonly used arguments for explaining gender differences in spatial tasks. However, this problem has not been verified at the neurological level. Therefore, the current research hopes to verify the above problems through study 2 and put forward the following research hypothesis:

H1. The neural basis of gender differences in spatial anxiety exists in individuals with large-scale spatial ability.
H2. The neural basis of gender differences in spatial anxiety exists in individuals with small-scale spatial ability.
H3. The neural basis of gender differences in cognitive strategies exists in individuals with large-scale spatial ability.
H4. The neural basis of gender differences in cognitive strategies exists in individuals with small-scale spatial ability.

## Methods

### Literature Search and Study Selection

Study 2 followed Study 1's logic in literature search and screening. Of all the literature retrieved and downloaded in the first step of Study 1's literature search, we first incorporated those using Functional Magnetic Resonance Imaging (FMRI) or Positron Emission Tomography (PET) technologies into our long list and then coded those meeting the conditions as follows:

1. All of the subjects are sample groups of healthy people.
2. Data analysis must adopt the whole brain analysis instead of the region of interest (ROI) analysis, and the data reported by the institute is standardized data (Montreal Neurological Institute-MNI or Talairach).
3. The specific experimental method in the study must be behavioral. And the articles must include reports of the males' and/or females' brain imaging data when they finish their independent experimental task, respectively.
4. If an experimental result is reported many times in multiple papers, but it has been recorded only once in the meta-analysis, the research data cannot be used again.

After the above screening process, there were totally 41 studies (Table 4; Figure 1), involving 677 participants (male = 447), that met the standards, and total of 1,366 foci were incorporated in the meta-analysis of this research. Among them, there were 467 foci associated with males' large-scale spatial ability, 51 foci associated with females' large-scale spatial ability, 517 foci related to males' small-scale space ability, and 331 foci related to females' small-scale space ability.

**Table 4:** Summary of studies included in the present meta-analyses of study 2.

**Study**	***N***	**Mean age**	**Country**	**Task and contrast**	**Foci**
**LARGE-SCALE SPATIAL ABILITY**					
**Males**					
Baumann et al., 2010	17	31.6	Australia	Navigation task > control, good navigators > poor navigators	64
Grön et al., 2000	24	26	Germany	Navigation task > control	18
Hartley and Maguire, 2003,	16	28.9	UK	Good navigation performance > poor navigation performance, wayfinding > trail following, wayfinding > route following	23
Ino et al., 2002	16	32.3	Japan	Mental navigation task > control	10
Ino et al., 2007	1	55	Japan	Navigation task > control	7
IglóiI et al., 2010	19	24.3	UK	Training trials in navigation > control trials, allocentric and egocentric responses trials > control trials, allocentric responses trials > egocentric responses trials	95
Lux et al., 2003	14	26.8	Germany	Spatial orientation task > control	10
Lee et al., 2005	10	22–25	Hong Kong	Spatial orientation task > control	39
Latini-Corazzini et al., 2010	16	21.2	Italy	Route task > control, survey task > control, route task > survey task	31
Maguire et al., 1997	11	45	UK	Routes, landmarks, film plots, and film frames tasks > control	35
Rosenbaum et al., 2004	10	26.4	Canadian	Mental navigation task > control	35
Wolbers and Büchel, 2005	11	19–28	Germany	Learning, performance, and change phase in navigation task > control	17
Xu et al., 2010	20	24.2	Norway	The conditions of Normal, Without and Blocked in navigation task > Line following	83
**Females**
Clemente et al., 2013	14	21.64	Spain	Navigation > video, navigation > photographs	10
Grön et al., 2000	12	26	Germany	Navigation task > control	18
Pintzka et al., 2016	53	22.5	Norway	Successful > failed navigation	23
**SMALL-SCALE SPATIAL ABILITY**					
**Males**					
Corradi-Dell'Acqua et al., 2009	17	28.31	Germany	Body schema and body structural rotation > control, stimulus strategy > control	5
De Lange et al., 2006	17	24	Netherlands	Mental rotation task > control	7
Halari et al., 2006	9	25.78	UK	Mental rotation task > control	27
Jordan et al., 2002	10	23.17	Germany	Three mental rotation conditions (3d, abstract, letter) > control, 3D-condition > the ABSTRACT- and LETTER-conditions	36
Kucian et al., 2005	22	25.9	Switzerland	Mental rotation task > control	37
Kawamichi et al., 2007	14	18–33	Japan	Mental rotation task > control	40
Lamm et al., 2001	13	24.5	Austria	Mental rotation task > control	11
Lange et al., 2005	6	25	Netherlands	Mental rotation task > control	10
Lamm et al., 2007	13	23–31	Austria	Location and orientation condition during mental rotation > control	16
Ng et al., 2001	12	29.25	UK	Line orientation experiment > control, mental rotation experiment > control	15
O'Boyle et al., 2005	6	14.3	Australia	Mental rotation task > control	7
Prescott et al., 2010	8	14.2	Australia	Mental rotation task > control	18
Paschke et al., 2012	10	25	Germany	Mental rotation task > control	3
Seurinck et al., 2004a	11	25.4	Belgium	Mental rotation task > control	46
Sluming et al., 2007	10	41	UK	Mental rotation task > control	14
Schöning et al., 2007	14	32	Germany	Mental rotation task > control	95
Seurinck et al., 2011	16	24	Netherlands	Mental rotation task > control	16
Stoodley et al., 2012	9	25	USA	Rotated letters > upright letters	18
Vingerhoets et al., 2002	13	29	Belgium	Rotated hands and figures > control	31
Wolbers et al., 2006	16	19–29	Germany	Spatial visualization task > control	6
Weiss et al., 2009	16	20–39	Germany	Mental rotation > stimulus categorization, mirrored presentation > non-mirrored presentation	59
**Females**					
Ecker et al., 2006	10	20–30	UK	Mental rotation task > control	15
Ebisch et al., 2012	22	20–24	Italy	Induction–visualization > induction–spatial relationships, visualization–induction > visualization–spatial relationships	8
Gogos et al., 2010	10	55.4	Australia	Mental rotation task > control	16
Halari et al., 2006	10	25.78	UK	Mental rotation task > control	27
Jordan et al., 2002	14	23.17	Germany	Three mental rotation conditions (3d, abstract, letter) > control, 3D-condition > the ABSTRACT- and LETTER-conditions	36
Kucian et al., 2005	12	25.9	Switzerland	Mental rotation task > control	37
Papeo et al., 2012	18	22–28	USA	Motor strategy and visuospatial strategy > control	15
Seurinck et al., 2004a	11	25.4	Belgium	Mental rotation task > control	46
Seurinck et al., 2004b	24	23	Belgium	Mental rotation task > control	36
Schöning et al., 2007	20	32	Germany	Mental rotation task > control	95

### Activation Likelihood Estimation

ALE is the most common algorithm for coordinate-based meta-analyses (Eickhoff et al., 2012). It treats activation foci reported in neuroimaging studies not as single points but as spatial probability distributions centered at the given coordinates. ALE maps are then obtained by computing the union of activation probabilities for each voxel. The current research is using the revised algorithm for ALE analysis which proposed by Eickhoff et al. (2009). It models the spatial uncertainty—and thus probability distribution—of each focus using an estimation of the inter-subject and inter-laboratory variability typically observed in neuroimaging experiments, rather than using a pre-specified full- width half maximum (FWHM) for all experiments as originally proposed. The modified permutation procedure reflects a null-distribution of a random spatial association between studies (i.e., random-effects analysis) not between foci (i.e., fixed-effects analysis; Eickhoff et al., 2016, 2017).

The ALE method may focus on resolving the following problems in the current brain imaging research: firstly, the number of subjects in the single brain imaging study is generally less, and the results are not stable enough; secondly, the result of a single brain imaging are probably affected by certain experimental operations (e.g., scan parameters); thirdly, the interpretation of the function of a certain brain region by a single brain imaging study is often limited to a single or a few tasks used.

The Ginger ALE software (version 2.3, http://www.brainmap.org/ale; Feng et al., 2015) were used to conducted the current meta-analysis (also include the conversion of Talairach coordinates into MNI). The resulting *p-*value maps were thresholded using the false discovery rate (FDR) correction at *p* < 0.05 with 5,000 threshold permutations (Genovese et al., 2002; Laird et al., 2005), and all clusters were set to a minimum volume of 600 mm^3^ (Lamm et al., 2011). The results were overlaid onto an anatomical template (Colin27 T1 seg MNI.nii; Luo et al., 2018) and displayed using the Mango software (http://rii.uthscsa.edu/mango; Feng et al., 2018).

## Results

**Figure 4:**
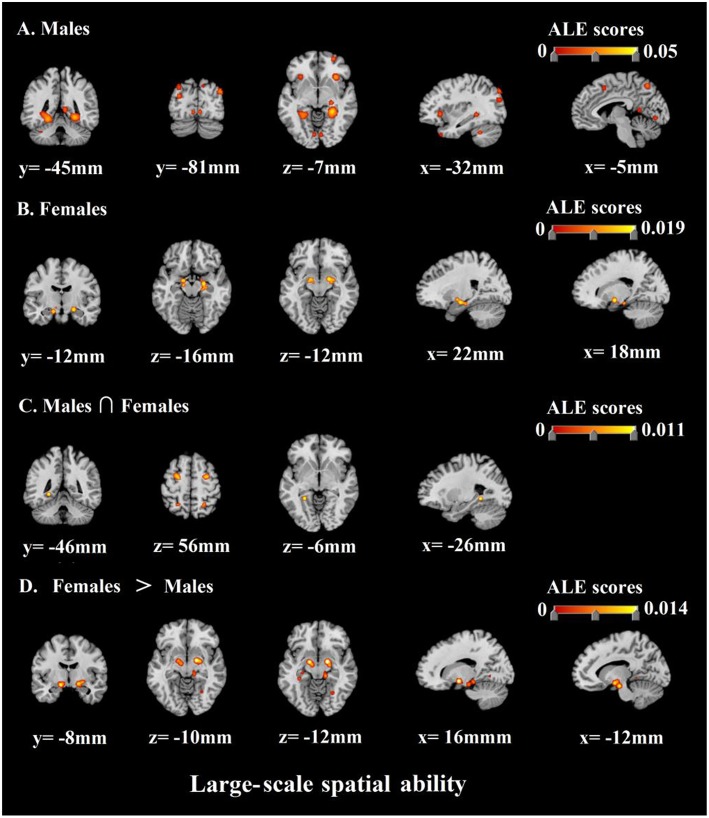
ALE meta-analysis of neuroimaging studies on males' **(A)** and females' **(B)** large-scale spatial ability. And the results of conjunction analysis and contrast analysis: **(C)** The common regions associated with males' and females' large-scale spatial ability; **(D)** Brain regions activated more by females than males.

In terms of the large-scale spatial ability, the results of the ALE analysis identified 25 clusters of consistent activation for males and 4 clusters females. The former clusters of males were mainly in bilateral limbic lobe, bilateral occipital lobe, bilateral sub-lobar, bilateral frontal lobe, left temporal lobe, left parietal lobe, bilateral anterior lobe, and left posterior lobe. The latter clusters of females were concentrated in bilateral limbic lobe and right sub-lobar. The specific areas of the individual focus see Table 5 and Figure 4. Furthermore, to explore common and distinct neural regions of males and females, we compared the above ALE meta-analytic results. The result of conjunction analysis revealed that left limbic lobe was activated in both of them. In addition, females and males contrast demonstrated correspondence in bilateral sub-lobar and bilateral limbic lobe. Conversely, no suprathreshold clusters were revealed by the males and females contrast (Table 6; Figure 4).

**Table 5:** ALE meta-analysis results for large-scale spatial ability.

**Cluster No**.	**Laterality**	**Brain regions**	**BA**	**x**	**y**	**z**	**ALE (10^**−**^^2^)**	**Cluster size (mm3)**
**Males**								
1	R	Parahippocampal Gyrus	36	26	−40	−10	0.050369	3288
2	L	Parahippocampal Gyrus	36	−22	−44	−12	0.034565	2200
	L	Parahippocampal Gyrus	19	−32	−42	−4	0.022634	
3	L	Posterior Cingulate	30	−14	−56	18	0.02931	1784
	L	Posterior Cingulate	29	−10	−52	8	0.020769	
4	R	Superior Occipital Gyrus	19	44	−78	32	0.030861	1064
5	R	Claustrum		32	24	−4	0.039246	1032
6	L	Medial Frontal Gyrus	32	0	12	48	0.029162	872
7	L	Insula	13	−32	24	−2	0.030118	736
8	R	Posterior Cingulate	30	16	−54	16	0.02585	664
9	L	Superior Temporal Gyrus	38	−42	18	−34	0.023836	496
10	L	Precuneus	7	−4	−66	54	0.023073	496
11	L	Middle Occipital Gyrus	19	−32	−84	24	0.024111	472
12	L	Superior Occipital Gyrus	19	−32	−86	40	0.024569	416
13	R	Superior Frontal Gyrus	10	28	56	−6	0.022647	384
14	R	Culmen		8	−46	4	0.019288	376
15	R	Parahippocampal Gyrus	28	24	−24	−8	0.020488	304
16	R	Caudate		18	28	8	0.017964	304
	R	Caudate		18	26	0	0.01794	
17	L	Culmen		−10	−70	−2	0.018203	288
18	R	Thalamus		8	−16	8	0.019477	232
19	R	Lingual Gyrus	18	6	−82	−6	0.01892	168
20	R	Inferior Frontal Gyrus	9	48	12	30	0.018163	168
21	L	Lingual Gyrus	18	−6	−80	−6	0.017503	144
22	L	Cerebellar Tonsil		−34	−50	−36	0.01747	136
23	R	Parahippocampal Gyrus		30	−6	−16	0.017945	136
24	L	Inferior Frontal Gyrus	6	−48	6	32	0.018083	136
25	R	Cuneus	19	12	−78	42	0.017716	136
**Females**								
1	R	Parahippocampal Gyrus		22	−12	−16	0.019123	1280
	R	Lentiform Nucleus		18	−6	−12	0.019109	
	R	Lentiform Nucleus		26	−6	−10	0.01542	
	R	Parahippocampal Gyrus	35	20	−22	−18	0.013516	
	R	Parahippocampal Gyrus	28	28	−20	−14	0.011108	
2	L	Parahippocampal Gyrus	28	−15	−6	−14	0.019109	464
3	L	Parahippocampal Gyrus	28	−14	−14	−18	0.01877	336
4	L	Parahippocampal Gyrus	37	−26	−46	−6	0.014915	208

*BA, Brodmann area; R, right; L, left*.

**Table 6:** ALE meta-analysis results of conjunction and contrast analysis of large-scale spatial ability of meals and females.

**Cluster No**.	**Laterality**	**Brain regions**	**BA**	**x**	**Y**	**z**	**ALE (10^**−**^^2^)**	**Cluster size (mm3)**
**Males∩Females**								
1	L	Parahippocampal Gyrus	37	−26	−46	−6	0.014915	184
**Males > Females**								
**None**								
**Females > Males**								
1	R	Lentiform Nucleus		16	−8	−10	0.01058	1776
	R	Lentiform Nucleus		14	−4	−12	0.01028	
	R	Parahippocampal Gyrus	28	20	−16	−16	0.00768	
	R	Parahippocampal Gyrus	28	18	−12	−16	0.0101	
	R	Lentiform Nucleus		23.6	−8.1	−10.2	0.01085	
2	L	Parahippocampal Gyrus	34	−14.8	−16	−20	0.0114	968
	L	Lentiform Nucleus		−12	−8	−12	0.01086	
	L	Lentiform Nucleus		−16	−10	−12	0.00733	
3	L	Parahippocampal Gyrus	19	−24	−46	−2	0.0054	424

**Figure 5:**
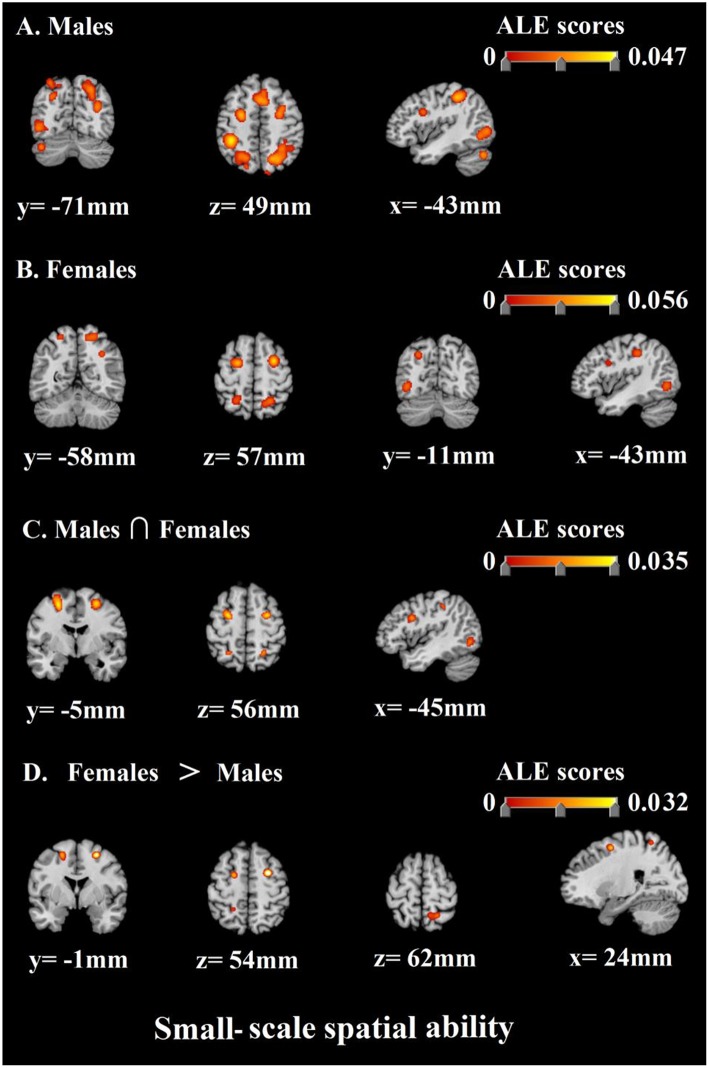
ALE meta-analysis of neuroimaging studies on males' **(A)** and females' **(B)** small-scale spatial ability. And the results of conjunction analysis and contrast analysis: **(C)** The common regions associated with males' and females' small-scale spatial ability; **(D)** Brain regions activated more by females than males.

Then, as to the small-scale spatial ability, the results of the ALE analysis identified 18 clusters of consistent activation for males and 13 clusters females (Table 7; Figure 5). The previous clusters of males were mainly in bilateral parietal lobe, bilateral frontal lobe, bilateral occipital lobe, bilateral posterior lobe, and left sub-lobar. The next clusters of females were concentrated in bilateral frontal lobe, bilateral parietal lobe, and bilateral occipital lobe. Besides, the result of conjunction analysis revealed that bilateral frontal lobe, left occipital lobe, and bilateral parietal lobe were activated in both of males and females. Finally, females and males contrast demonstrated correspondence in bilateral frontal lobe and right parietal lobe. Similarly, no suprathreshold clusters were revealed by the males and females contrast (Table 8; Figure 5).

**Table 7:** ALE meta-analysis results for small-scale spatial ability.

**Cluster No**.	**Laterality**	**Brain regions**	**BA**	**X**	**Y**	**z**	**ALE (10^**−**^^2^)**	**Cluster size (mm3)**
**Males**								
1	R	Precuneus	7	24	−68	46	0.034162	10360
	R	Precuneus	7	26	−60	54	0.033126	
	R	Precuneus	7	30	−52	54	0.031728	
	R	Precuneus	7	34	−40	42	0.026622	
	R	Superior Parietal Lobule	7	42	−52	54	0.023221	
	R	Supramarginal Gyrus	40	40	−40	34	0.017652	
2	L	Inferior Parietal Lobule	40	−38	−40	48	0.046883	8664
	L	Superior Parietal Lobule	7	−22	−62	54	0.035032	
	L	Superior Parietal Lobule	7	−38	−52	62	0.017468	
	L	Precuneus	7	−18	−76	50	0.014879	
3	L	Precentral Gyrus	6	−26	−6	54	0.040707	3648
4	L	Medial Frontal Gyrus	32	2	14	48	0.02908	3280
	R	Medial Frontal Gyrus	6	6	22	46	0.022784	
5	L	Occipital Gyrus	19	−40	−80	2	0.026659	2880
	L	Fusiform Gyrus	19	−46	−72	−6	0.022888	
6	R	Middle Frontal Gyrus	6	28	−6	56	0.035374	2872
	R	Middle Frontal Gyrus	6	30	2	48	0.02338	
	R	Middle Frontal Gyrus	6	34	4	62	0.015455	
7	L	Inferior Frontal Gyrus	9	−46	8	26	0.025227	1856
8	R	Tuber		42	−62	−32	0.032616	1368
9	R	Inferior Frontal Gyrus	9	58	14	24	0.022069	1312
	R	Middle Frontal Gyrus	9	56	10	36	0.019196	
10	R	Precuneus	31	34	−72	22	0.024916	1192
11	L	Pyramis		−42	−74	−32	0.029963	656
12	L	Insula	13	−32	26	−2	0.023349	656
13	L	Precuneus	19	−28	−72	36	0.020124	544
14	R	Inferior Frontal Gyrus	47	32	30	−8	0.020464	376
15	R	Middle Occipital Gyrus	18	46	−82	−8	0.019319	312
16	R	Middle Frontal Gyrus	46	44	40	14	0.018787	272
17	R	Cuneus	19	10	−86	46	0.018375	240
18	R	Postcentral Gyrus	2	56	−22	38	0.015817	112
**Females**								
1	L	Middle Frontal Gyrus	6	−22	−4	54	0.04258	2464
2	R	Sub-Gyral	6	28	0	54	0.055919	1984
3	R	Precuneus	7	22	−54	60	0.031903	1648
4	L	Inferior Occipital Gyrus	19	−42	−72	−4	0.024078	1360
	L	Lingual Gyrus	18	−34	−78	−2	0.017991	
5	L	Inferior Parietal Lobule	40	−46	−30	40	0.021931	1096
6	L	Inferior Frontal Gyrus	9	−48	8	28	0.024503	984
	L	Inferior Frontal Gyrus	9	−50	14	20	0.01735	
7	R	Inferior Occipital Gyrus	18	34	−82	0	0.024498	920
8	R	Inferior Parietal Lobule	40	38	−42	46	0.01945	904
9	L	Precuneus	7	−26	−68	38	0.025225	856
10	L	Precuneus	7	−24	−56	60	0.018502	808
	L	Precuneus	7	−20	−50	54	0.017932	
11	R	Inferior Frontal Gyrus	9	52	8	24	0.020205	704
	R	Precentral Gyrus	6	46	6	30	0.01523	
12	R	Precuneus	7	32	−60	38	0.018497	312
13	R	Inferior Frontal Gyrus	44	62	14	16	0.018392	264

**Table 8:** ALE meta-analysis results of conjunction and contrast analysis of small-scale spatial ability of meals and females.

**Cluster No**.	**Laterality**	**Brain regions**	**BA**	**x**	**y**	**z**	**ALE (10^**−**^^2^)**	**Cluster size (mm3)**
**Males∩females**								
1	L	Middle Frontal Gyrus	6	−24	−4	54	0.034997	1576
2	R	Middle Frontal Gyrus	6	28	−4	56	0.030599	1080
3	L	Fusiform Gyrus	19	−42	−70	−6	0.020082	888
	L	Inferior Occipital Gyrus	19	−36	−78	0	0.017696	
4	L	Inferior Frontal Gyrus	9	−48	8	28	0.023748	720
5	R	Inferior Parietal Lobule	40	38	−42	44	0.017832	408
6	R	Precuneus	7	26	−54	58	0.021182	408
7	L	Precuneus	19	−26	−72	38	0.018395	272
8	L	Precuneus	7	−24	−56	58	0.017343	208
9	L	Inferior Parietal Lobule	40	−42	−34	42	0.017033	184
10	R	Inferior Frontal Gyrus	9	56	10	24	0.01525	176
**Males > Females**								
**None**								
**Females > Males**								
1	R	Sub-Gyral	6	24.4	1.9	55.3	0.03247	800
2	R	Precuneus	7	15.9	−53.1	61.8	0.01462	576
3	L	Middle Frontal Gyrus	6	−17.8	−1.1	53.8	0.02327	552

## Discussion 2

Firstly, we discovered that when males were accomplishing large-scale spatial tasks, the areas with higher communalities in the activated brain regions included bilateral parahippocampal gyrus, bilateral posterior cingulate, bilateral superior occipital gyrus, right claustrum, left medial frontal gyrus, left insula, left superior temporal gyrus, left precuneus, left middle occipital gyrus, right superior frontal gyrus, bilateral culmen, right caudate, right thalamus, bilateral lingual gyrus, bilateral inferior frontal gyrus, left cerebellar tonsil, and right cuneus. Correspondingly, when females were accomplishing large-scale spatial tasks, the areas with higher consistency in the activated brain regions included bilateral parahippocampal gyrus and right lentiform nucleus. In conclusion, the above-mentioned brain regions are important neural basis for the large-scale spatial ability of males and females.

Besides, the most important work of this research is to compare the similarity and difference of neural activity in the course of cognitive processing of large-scale spatial information of males and females by the method of ALE. As shown by the results, we found that males and females still had a neural basis with higher communalities when completing large-scale spatial tasks. The results of conjunction analysis prove that left parahippocampal gyrus participate in the cognitive processing of large-scale spatial information of both males and females. This result indicates that the parahippocampal gyrus is the core of the neural basis of large-scale spatial ability no matter for males or for females.

What's more, we not only analyzed the neural basis shared by the males' and females' large-scale spatial ability, but we also analyzed and reported the specific neural activities they involved. Specifically, on the one hand, we discovered through the analysis of the contrast of females and males, that when females executed large-scale spatial tasks, the bilateral lentiform nucleus and bilateral parahippocampal gyrus were activated more intensely. Among them, lentiform nucleus was extensively proved to be related to the individual's emotional experience. Moreover, lentiform nucleus had a close link to individual negative emotions, which is also consistent with the findings of previous studies (Goldin et al., 2008; Telzer et al., 2014; Wardle et al., 2014; Touroutoglou et al., 2015). It shows that females experience more negative emotions (spatial anxiety) than males when performing large-scale spatial tasks. This result is consistent with previous studies on many behavioral levels (Lawton, 1994; Malinowski and Gillespie, 2001; Lawton and Kallai, 2002; Gabriel et al., 2011). Gabriel et al. (2011)'s study proposed that the key to gender differences in individual spatial ability was the time limit for experimental tasks. The time limit puts females under great pressure so that they feel fear and anxiety, and such negative emotions are the direct cause of males outperforming females. Our study further verified Gabriel et al. (2011)'s theory with evidence of its neural basis. Meanwhile, our findings support the research hypothesis 1. In detail, in large-scale spatial tasks, female individuals exhibit stronger neural activity related to spatial anxiety than male individuals.

In addition, as mentioned above, parahippocampal gyrus is the main neural basis of individual large-scale spatial ability. The interesting thing to note is that males still outperformed females in large-scale spatial tasks even though females were better activated in parahippocampal gyrus, which probably means their parahippocampal gyrus works less efficiently than males. On the other hand, nevertheless, the analysis of the contrast between males and females found no specific brain regions in males, showing that males have no specific neural basis for cognitive processing of large-scale spatial information. In other words, it can be considered as, the contrast between males and females studies failed to show any suprathreshold cluster, demonstrating that males' spatial ability recruits a subset of areas also by females' spatial ability. In view of this, we believe behavioral gender differences in large-scale spatial ability are mainly due to the specific neural basis of females rather than males.

Secondly, corresponding to the above gender differences in large-scale spatial ability. we also discovered that when males were accomplishing small-scale spatial tasks, the areas with higher communalities in the activated brain regions included bilateral precuneus, bilateral superior parietal lobule, right supramarginal gyrus, left inferior parietal lobule, left precentral gyrus, bilateral medial frontal gyrus, left occipital gyrus, left fusiform gyrus, bilateral inferior frontal gyrus, right tuber, right middle frontal gyrus, left pyramis, left insula, right middle occipital gyrus, right cuneus, and right postcentral gyrus. Correspondingly, when females were accomplishing small-scale spatial tasks, the areas with higher consistency in the activated brain regions included left middle frontal gyrus, right sub-gyral, bilateral precuneus, bilateral inferior occipital gyrus, left lingual gyrus, bilateral inferior parietal lobule, bilateral inferior frontal gyrus, and right precentral gyrus. In general, the above-mentioned brain regions are important neural basis for the small-scale spatial ability of males and females.

Then, similarly, the next most important work of this research is to compare the similarity and difference of neural activity in the course of cognitive processing of small-scale spatial information of males and females. As shown by the results, we found that males and females still had a neural basis with higher communalities when completing small-scale spatial tasks. The results of conjunction analysis prove that bilateral middle frontal gyrus, left fusiform gyrus, left inferior occipital gyrus, bilateral inferior frontal gyrus, bilateral inferior parietal lobule, and bilateral precuneus participate in the cognitive processing of small-scale spatial information of both males and females. This result indicates that the above-mentioned brain regions are important neural basis for individual small-scale spatial ability no matter for males or for females.

In addition, the analysis of the contrast between females and males showed that compared with males, females were stronger activated in right sub-gyral, right precuneus, and left middle frontal gyrus during cognitive processing of small-scale spatial information, which corresponds to the above gender differences in large-scale spatial ability. Right sub-gyral is the key brain region for the individual to perform executive control (Kerstin et al., 2005; Schubotz and von Cramon, 2009; Fan et al., 2012; Dambacher et al., 2013). Butler et al. (2006) said females made more efforts in top-down executive control than males and were thus better activated in brain regions like sub-gyral when performing cognitive processing of spatial information, which is in line with our findings. Besides, precuneus and middle frontal gyrus are also proved to be key brain regions reflecting individual adoption of the egocentric strategy (Galati et al., 2000). This finding also supports our research hypothesis 4. The allocentric and the egocentric are the two most commonly used strategies for individuals to perform cognitive processing of spatial information. A lot of researchers hold that the egocentric strategy is more suitable for individuals to perform large-scale spatial tasks while the allocentric strategy is more suitable in small-scale spatial tasks (Malinowski, 2001; Zacks et al., 2001; Peña et al., 2008). It can therefore be inferred that behavioral gender differences in small-scale spatial ability may also be associated with cognitive strategies adopted by individuals; that is to say, females tend to adopt the egocentric strategy when performing small-scale spatial tasks, whereas the allocentric strategy is the best choice for individuals to perform such spatial tasks. It is precisely the failure to make the best choice that leads to the inferior performance of females. Meanwhile, it is noteworthy that all our discussions are based on the premise of “most females.” In fact, there is no absolute difference and boundary in individual cognitive strategies whether between males and females or between large-scale and small-scale spatial tasks, which makes it possible that both males and females choose either strategy when it comes to large-scale or small-scale spatial tasks. Furthermore, on the other side, in terms of small-scale spatial ability, the analysis of the contrast of males and females saw no specific brain regions in males, which is similar to the result of the large-scale spatial ability analysis. It shows that males have no specific neural basis for cognitive processing of small-scale spatial information. Similarly, we believe behavioral gender differences in small-scale spatial ability are mainly due to the specific neural basis of females rather than males.

## General Discussion

We first revealed the different behavioral manifestations of individual gender differences in large-scale and small-scale Spatial Ability through Study 1. We then analyzed the potential neural basis of such behavioral differences through Study 2. On the behavioral level, we found in Study 1 individuals showed a high level of gender differences in large-scale spatial ability and a medium level of gender differences in small-scale spatial ability. Although males outperformed females in both large-scale and small-scale Spatial Ability, this gender gap is significantly smaller in small-scale spatial ability than in large-scale spatial ability.

On the level of neural basis, we found in Study 2 that males and females shared a common neural basis in both large-scale and small-scale Spatial Ability. The different thing is that compared with large-scale spatial ability there are more overlapping brain regions in small-scale spatial ability, which means a broader neural basis shared by males and females. We believe this is also one of the reasons for different behavioral manifestations of gender differences in large-scale and small-scale Spatial Ability. At the same time, no specific brain regions were found in males in both Spatial Ability, while females showed some specific brain activities. It should be noted, however, that the specific brain activities of females manifested completely differently in large-scale and small-scale Spatial Ability. This also suggests that although females performed not so well as males in both Spatial Ability, the reasons for such performance were completely different. That is to say, the reason why females performed not so well in large-scale spatial ability was that they were more susceptible to spatial anxiety and their parahippocampal gyrus worked less efficiently than males; females performed not so well in small-scale spatial ability because they mostly adopted the egocentric strategy and their sub-gyral also worked less efficiently than males. The two different reasons have made for gender differences in favor of males in terms of spatial ability and such gender differences have different manifestations in large-scale and small-scale Spatial Ability.

To sum up, we believe that behavioral gender differences in large-scale and small-scale Spatial Ability are mainly due to different neural bases. But what makes for such difference in the neural basis? The evidence in this regard is still inconclusive. A great many researchers have offered their thoughts, hypotheses, and explanations. Some hypotheses were made from the perspective of evolutionary psychology: (1) Dispersal Hypothesis- Natal dispersal distance varies between sexes. (2) Fertility and parental care Hypothesis- Females reduce mobility to decrease mortality during reproductive periods. (3) Male foraging Hypothesis- ivision of foraging labor: Men use navigation skills for hunting. (4) Range size- Polygynous males have larger ranges to mate with more females. (5) Male warfare- Men travel long distances to kill competitors and capture females. (6) Female choice- Women choose males on the basis of their hunting success (Jones et al., 2003). The most widely recognized one is the Male foraging Hypothesis, also known as the Hunter-Gatherer Theory (HGT; Silverman et al., 2007; Burke et al., 2012). The hunter-gatherer is a human living in a society in which most or all food is obtained by foraging (collecting wild plants and pursuing wild animals). Hunting and gathering was humanity's first and most successful adaptation, occupying at least 90 percent of human history (Little, 2016). Accordingto the HGT, sex-specific patterns of spatial behavior emerged with the appearance of a hunter-gatherer way of life, accompanied by a sexual division of labor. Men in prehistory are assumed to have been predominantly hunters, ranging widely in unfamiliar surroundings (Burke et al., 2012). In a nutshell, such long-term learning, practice, reinforcement, experience, and evolution are responsible for gender differences in individual spatial ability today.

Of course, in addition to the above factors, there are also possible reasons for the current gender differences in individual spatial ability is social factors. For both children and adults, female individuals and male individuals are given different social expectations in social development. This social expectation has a subtle influence on the performance of female and male individuals in many social activities, such as the choice of toys or games (Raag and Rackliff, 1998; Raag, 1999), social division of labor (Kluwer and Mikula, 2003), learning and scientific research (Hirshfield, 2014). Under the influence of social expectations, individuals' continuous adaptation, and long-term practice of the above social activities may cause and exacerbate the existing gender differences in such individual spatial abiliy. And these gender differences will in turn affect the individual's subsequent social activities, and so on. For example, According to the statistics of Hoffman et al. (2011)'s study, In the general science, engineering, and technology industries, the number of male labor is four times that of female. In the more prominent areas of research of math, chemistry, physics, and mechanical engineering, male tenured professors outnumber female tenured professors 8 to 15 times. Of course, there are many similar data. Hirshfield (2014) believes that the reason for this situation is that female individuals face many obstacles that male individuals do not face in science, technology, engineering, and mathematics (STEM) educational programmes and careers. Such as differential pay, chilly departmental or workplace climates, a greater likelihood of leaving STEM programmes or careers, better to be an adjunct faculty, and family pressures. Hirshfield (2014) also points out that female individuals often face the following three kinds of prejudicial social expectations in STEM educational programmes and careers: science is associated with masculinity, being a professor is also associated with being a man, and leadership is often associated with men and masculinity and as a result. These social expectations may have a negative impact on females' study and work in STEM and other fields. And the lack of adequate STEM training may lead to a gap between females and males in terms of spatial ability. We can't say that such gender differences are mainly because females are inferior to males in spatial ability, but we believe individual spatial ability is at least one of the contributing factors. Of course, the causes of gender difference in individual spatial ability are complex and long-term, and it cannot be effectively solved in one or two aspects in a short time. As far as the current research is concerned, we hope to provide more reference and inspiration for understanding and solving the gender differences in individual spatial ability from the cognitive and neural basis levels. As spatial ability is inextricably linked to the above disciplines and fields, we hope our study can offer a new perspective to enhance women's spatial ability as well as a targeted, viable approach to that end, examples of which can be training women in control and alleviate spatial anxiety or improving cognitive strategy, training individual parahippocampal gyrus by use of transcranial magnetic stimulation (TMS), and so forth. Spatial ability training and transfer as such are exactly what we hope to study in the future.

Several limitations of the current study should be noted. Firstly, the quantity of researches on large-scale spatial ability in study 1 as well as study 2, especially for females, is not abundant. Secondly, unlike meta-analyses used in other fields of research, the ALE calculations based on neuroimaging do not consider the size of an effect; consequently, they cannot include evidence for the absence of an effect, so-called null results. And the ALE also cannot illuminate the temporal dynamics of cognitive processes (Winlove et al., 2018).

## Conclusion

To summarize, the main work of this study is that we find that although males outperform females in both large-scale and small-scale spatial ability, individuals show a high level of gender differences in large-scale spatial ability and a medium level of gender differences in small-scale spatial ability within acceptable publication bias.Then, we also discover that campared to meals, females demonstrate a stronger activation in bilateral lentiform nucleus and bilateral parahippocampal gyrus in the large-scale spatial task, and activation in right sub-gyral, right precuneus, and left middle frontal gyrus in small-scale spatial task. We belive that the reason why females perform not so well in large-scale spatial ability is that they are more susceptible to emotions and their parahippocampal gyrus work less efficiently than males; females perform not so well in small-scale spatial ability because they mostly adopt the egocentric strategy and their sub-gyral also work less efficiently than males. This also suggests that the above tow different reasons lead to the different behavioral performance of gender differences in large- and small-scale spatial ability.

## Data Availability

All datasets generated for this study are included in the manuscript and/or the Supplementary Files.

## Author Contributions

LY, FK, YL, SZ, JL, and XY: Design of the study, data collection, data analysis, paper writing and revising.

### Conflict of Interest Statement

The authors declare that the research was conducted in the absence of any commercial or financial relationships that could be construed as a potential conflict of interest.
